# Coronary heart disease and mortality following a breast cancer diagnosis

**DOI:** 10.1186/s12911-020-1127-y

**Published:** 2020-05-13

**Authors:** Aixia Guo, Kathleen W. Zhang, Kristi Reynolds, Randi E. Foraker

**Affiliations:** 1 Institute for Informatics (I2), Washington University School of Medicine, 600 S. Taylor Avenue, Suite 102, Campus Box 8102, St. Louis, MO 63110 USA; 2 Cardiovascular Division, Department of Internal Medicine, Washington University School of Medicine, St. Louis, MO USA; 3 Department of Research and Evaluation Southern California Permanente Medical Group, Pasadena, California USA; 4 Department of Health Systems Science, Kaiser Permanente School of Medicine, Pasadena, California USA; 5 Division of General Medical Sciences, Department of Internal Medicine, Washington University School of Medicine, 600 S. Taylor Avenue, Suite 102, Campus Box 8102, St. Louis, MO 63110 USA

**Keywords:** Cancer informatics, Machine learning, Precision medicine, Coronary heart disease, Death, Breast Cancer, Cancer treatments, Interactions

## Abstract

**Background:**

Coronary heart disease (CHD) is a leading cause of morbidity and mortality for breast cancer survivors, yet the joint effect of adverse cardiovascular health (CVH) and cardiotoxic cancer treatments on post-treatment CHD and death has not been quantified.

**Methods:**

We conducted statistical and machine learning approaches to evaluate 10-year risk of these outcomes among 1934 women diagnosed with breast cancer during 2006 and 2007. Overall CVH scores were classified as poor, intermediate, or ideal for 5 factors, smoking, body mass index, blood pressure, glucose/hemoglobin A1c, and cholesterol from clinical data within 5 years prior to the breast cancer diagnosis. The receipt of potentially cardiotoxic breast cancer treatments was indicated if the patient received anthracyclines or hormone therapies. We modeled the outcomes of post-cancer diagnosis CHD and death, respectively.

**Results:**

Results of these approaches indicated that the joint effect of poor CVH and receipt of cardiotoxic treatments on CHD (75.9%) and death (39.5%) was significantly higher than their independent effects [poor CVH (55.9%) and cardiotoxic treatments (43.6%) for CHD, and poor CVH (29.4%) and cardiotoxic treatments (35.8%) for death].

**Conclusions:**

Better CVH appears to be protective against the development of CHD even among women who had received potentially cardiotoxic treatments. This study determined the extent to which attainment of ideal CVH is important not only for CHD and mortality outcomes among women diagnosed with breast cancer.

## Background

Coronary heart disease (CHD) is the leading cause of death among all women [1], including breast cancer survivors [2–4]. Increased utilization of screening and treatment has led to more than 3.5 million female breast cancer survivors in the United States today [5, 6]. The majority of these women are more likely to die of CHD than cancer [2–4, 7, 8]. CHD is a serious issue, because important risk factors, such as physical inactivity, unhealthy diet, obesity, and smoking, are common to the etiology of both CHD and breast cancer [1, 9–11].

Cardiovascular health (CVH), as defined recently by the American Heart Association (AHA), has important implications for the prevention of both CHD and cancer [12, 13]. CVH factors are believed to operate in common pathways to chronic disease. For example, adverse CVH factors may be pro-inflammatory and also may be carcinogenic. To date, many community-based studies have used the CVH metric to characterize the prevalence of ideal CVH in population-based samples [14–19]. Our previous work in the Women’s Health Initiative (WHI) found that a poorer ideal CVH score, comprising the aforementioned factors plus blood pressure, cholesterol, and glucose, was associated with a higher incidence of cardiovascular disease, cancer, and breast cancer specifically [20].

Our evaluation of California cancer registry data highlighted the possible role of shared risk factors in the development of both cancer and CHD, reporting that cancer survivors tend to have multiple CHD risk factors, and that survivorship care often does not address these risk factors [21, 22]. Favorable levels of risk factors common to both CHD and cancer are associated with improved CHD and cancer survival [23]. Yet, in addition to the problem of shared risk factors, therapies used to treat breast cancer are linked with cardiovascular injury, thus increasing CHD susceptibility via the “multiple-hit” hypothesis [24–33]. Breast cancer therapies that are potentially cardiotoxic include chemotherapies, radiotherapy, hormonal treatments, and monoclonal antibodies [24].

To our knowledge, existing studies have not yet assessed the joint effect (interaction) of predisposing cardiovascular risk factors and cancer treatments among breast cancer survivors. Subpopulations, such as breast cancer survivors in poor CVH prior to their cancer diagnosis, may be particularly susceptible to the late effects of chemotherapy, radiation, and other cancer treatments. Thus, this analysis will build on our previous work in the WHI which assessed the relationship between CVH and incident CHD and cancer [20].

A better understanding of synergistic associations between poor CVH and breast cancer treatments on CHD risk after breast cancer has the potential to guide CHD and cancer treatment, as well as post-treatment cancer-related follow-up care is warranted. Screening and treatment of poor CVH at the time of cancer diagnosis and treatment planning may improve morbidity and mortality from CHD among breast cancer survivors [4, 21, 34–36]. Existing literature indicates that left-sided radiation, in certain doses, has a synergistic effect with pre-existing cardiac risk factors on the risk of ischemic heart disease [17]. Our goal was to add to this literature by investigating the receipt of radiation alongside other types of cancer therapies on risk of CHD and mortality using novel statistical techniques [37].

## Methods

### Data source and study design

In this study, electronic health record (EHR) data was obtained from a large midwestern medical center. The patients (*n* = 1934) were all initially diagnosed with breast cancer during 2006 or 2007 and did not have pre-existing CHD. We included follow-up data for 10 years following the initial diagnosis. Our goal was to investigate the association between CVH, potentially-cardiotoxic cancer treatments, age, race, and the 10-year risk of post-treatment CHD [38] and death, respectively. We defined CHD according to 217 unique ICD 9/10 diagnosis codes and 14 unique procedure codes, and date of death was ascertained from EHR data which were updated regularly with data from the National Death Index.

We utilized measures of CVH as follows: smoking status, body mass index (BMI), blood pressure, glucose/hemoglobin A1c, and cholesterol [20], which were introduced by the AHA and shown in detail in Table 1. The most recent CVH data were ascertained within the 5 years prior to the diagnosis of breast cancer. We used these baseline data to assign a pre-treatment CVH score to each woman with a breast cancer diagnosis. A value of “ideal” corresponded with 2 points on that submetric; a value of “intermediate” with 1 point; and a value of “poor” with 0 points.

**Table 1:** Measures of CVH in the EHR (Adapted from Lloyd-Jones, 2011) [38]

	Poor Health	Intermediate Health	Ideal Health
Health Behaviors			
Smoking status	Yes	Former ≤12 months	Never or quit > 12 months
Body mass index	≥ 30 kg/m^2^	25–29.9 kg/m^2^	< 25 kg/m^2^
Health Factors			
Total cholesterol	≥ 240 mg/dL	200–239 mg/dL or treated to goal	< 200 mg/dL
Blood pressure	Systolic ≥140 mmHg or Diastolic ≥90 mmHg	Systolic 120–139 mmHg or Diastolic 80–89 mmHg or treated to goal	Systolic < 120 mmHg Diastolic < 80 mmHg
Fasting plasma glucose	≥ 126 mg/dL	100–125 mg/dL or treated to goal	< 100 mg/dL

For smoking status, for which data were complete, we classified current smoking as 0 points, and not current smoking as 1 point, as there were no data available to indicate if they had never smoked or had quit for more than 1 year (representing the “ideal” category). However, not all CVH data were completed for all women; therefore, we imputed a value of 2 points for all missing submetric values. We tested the robustness of this strategy by imputing a value of 1 or 0, respectively, for missing values. For all analyses, overall CVH was calculated by a sum of all points, divided by the total possible points [10], and multiplied by 100, and was defined as: 0- < 30% for poor; 30- < 80% for intermediate; and 80–100% as ideal.

Of interest for these analyses were the following treatments, due to their potential adverse effects on the myocardium: chemotherapy, left-sided radiation, hormone-related or anti-estrogen pills, and Herceptin. We categorized the breast cancer treatments as eight categories according to what medicines were ordered: anthracyclines, hormone therapy, aromatase inhibitors, monoclonal antibodies, antimicrotubule agents, alkylating agents, antimetabolites, and other (e.g., Bortezomib). In our current analysis, we included the receipt (yes/no) of each type of treatment.

### Statistical analysis

We classified age into three groups: 20–40, 41–60, and 61–100 years. The age is the age at the breast cancer diagnosis. Race/ethnicity was defined as: black, non-black, and unknown. After we quantified and categorized the features of CVH, cancer treatments, age, and race, we applied both traditional statistical methods and machine learning algorithms [i.e., support vector machines (SVM) [39], decision tree [40], and logistic regression [41]] to investigate the associations between age, race, CVH, cancer treatments, the interaction between CVH, cancer treatments, and CHD and all-cause mortality, respectively. We also conducted the Welch’s t-test and produced boxplots to evaluate the differences between independent and joint effects of CVH and cancer treatments. In the machine learning models, we used CVH, treatment and the interaction of CVH and treatment as features, and applied linear SVM, decision tree, and logistic regression models, respectively, to predict if a woman had incident CHD or death during 10 years of follow-up. For the death prediction, we randomly selected a similar number of patients who had died (*n* = 468) to compare to a sample of patients who had not died (*n* = 374) due to the imbalance of our data according to mortality. We used all patient observations in the CHD risk prediction models. We tested the 5 CVH submetrics and 8 treatment categories as input features for the classification models. The dataset was randomly split into training (80%) and test (20%) data sets, on which the models were trained and then applied. Criteria of accuracy and area under the receiver operator curve (AUC) were calculated to evaluate the performance of the models. Analyses were conducted by using the libraries of Scikit-learn, Scipy, Matplotlib with Python, version 3.6.5 (2018).

## Results

The average age of the population was 58.5 years, and the majority of women (73%) were non-black (Table 2). Approximately 20% of women were currently taking a cholesterol medication, and few women (3%) were current smokers (Table 2). There were 341 patients with receipt of any class of cardiotoxic cancer treatments. Among these 341 patients, 46% women received aromatase inhibitors and 26% women received hormone therapy. During the 10-year follow-up period, one-third of the population developed CHD and 19% died.

**Table 2:** Characteristics [mean (SD) or n (%)] of the study population (*n* = 1934)

	Total (***n*** = 1934)	CHD(***n*** = 664)	Death (***n*** = 374)
Age (years)			
Mean (SD)	58.5 (12.9)	62.9 (13.1)	61.4 (13.9)
20–40	135 (7.0)	23 (3.5)	23 (6.1)
> 40–60	975 (50.4)	260 (39.2)	159 (42.5)
> 60–100	824 (42.6)	381 (57.4)	192 (51.3)
Race			
Black	435 (22.5)	209 (31.5)	126 (33.7)
Non-black	1419 (73.4)	433 (65.2)	226 (60.4)
Unknown	80 (4.1)	22 (3.3)	22 (5.9)
BMI (kg/m^2^)	21.9 (13.6)	29.3 (8.3)	28.1 (10.1)
Systolic blood pressure (SBP, mmHg)	125.4 (20.7)	128.4 (22.0)	126.0 (20.7)
Diastolic blood pressure (DBP, mmHg)	69.8 (11.2)	69.8 (11.6)	69.4 (11.4)
Fasting glucose (mg/dL)	109.3 (33.3)	129.9 (53.6)	127.6 (58.3)
Hemoglobin A1c (%)	6.94 (1.9)	7.05 (1.8)	7.45 (2.3)
Total cholesterol (mg/dL)	186.6 (50.3)	181.6 (46.6)	178.9 (61.0)
Current smoking	66 (3.4)	51 (7.7)	18 (4.8)
Taking antihypercholesterolemia medication	367 (19.0)	267 (40.2)	88 (23.5)
Taking antihypertensive medication	461 (23.8)	386 (58.1)	215 (57.5)
Taking diabetic medications	346 (17.9)	238 (35.8)	138 (36.9)
Classes of potentially cardiotoxic cancer treatments^a^			
Total patients (%) receiving treatments	341 (17.6)	201 (30.3)	128 (34.2)
Anthracyclines	6 (1.8)	4 (2.0)	3 (2.3)
Hormone therapy	87 (25.5)	51 (25.4)	28 (21.9)
Aromatase inhibitors	158 (46.3)	97 (48.3)	44 (34.3)
Monoclonal antibodies	7 (2.1)	3 (1.5)	2 (1.6)
Antimicrotubule agents	15 (4.4)	9 (4.5)	9 (7.0)
Alkylating agents	21 (6.2)	12 (6.0)	12 (9.4)
Antimetabolites	37 (10.9)	20 (10.0)	27 (21.1)
Other	10 (2.9)	5 (2.5)	3 (2.3)

^a^The percentages for each class of treatments used 341, 201 and 128 as the denominator

**Fig. 1:**
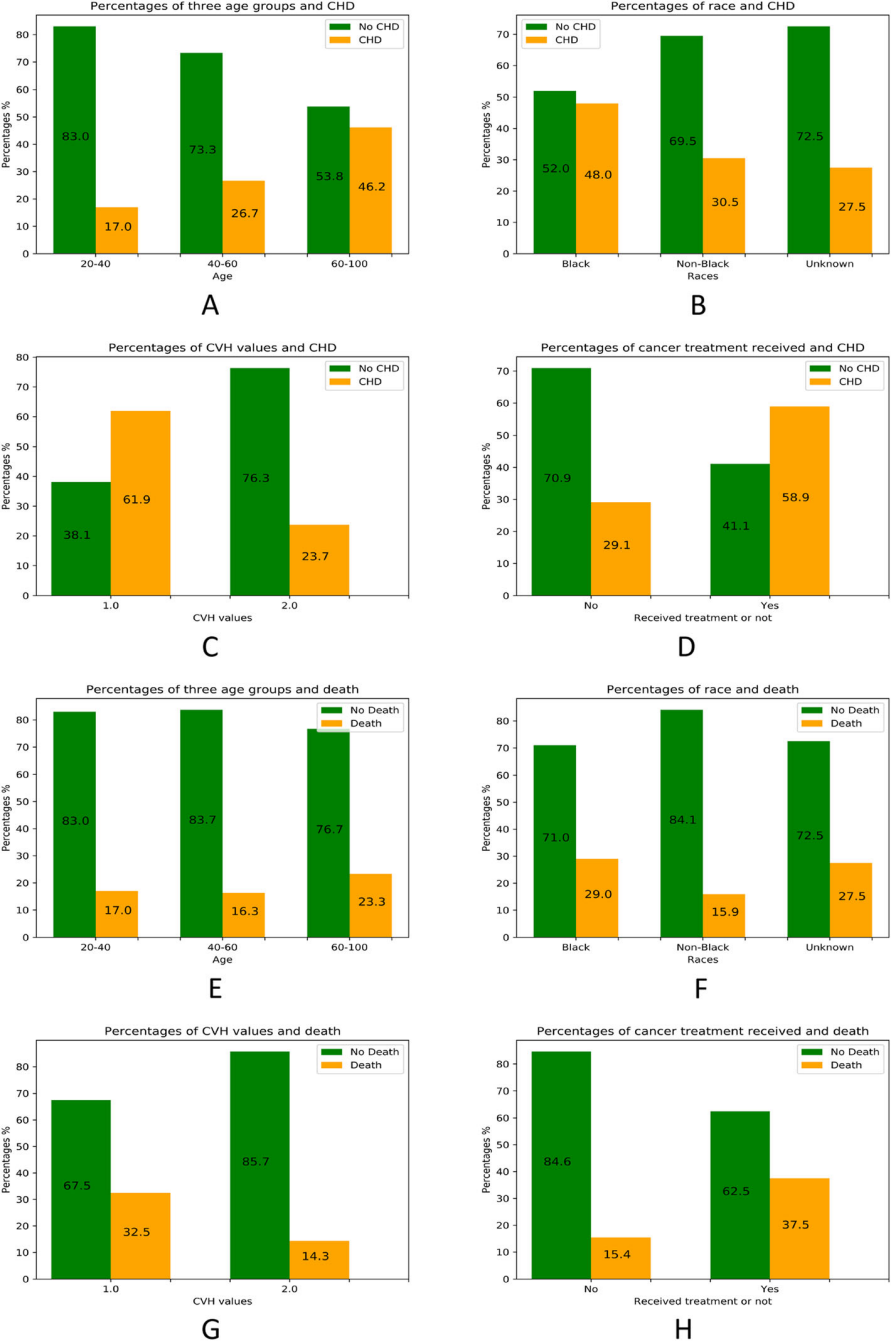
Associations between age, race, CVH, treatment and CHD or death. **a-d** show associations between age (**a**), race (**b**), CVH (**c**), treatment (**d**) and CHD. **e-h** show associations between age (**e**), race (**f**), CVH (**g**), treatment (**h**) and death. In **c** and **g**, CVH is ideal if CVH = 2.0, and intermediate if CVH = 1.0

Figure 1 shows the counts of women with each outcome of interest and the proportion of women represented in that strata. Women with a lower occurrence of CHD were younger (20–40 years), and the prevalence of CHD steadily increased across older age groups (Fig. 1a). Black women experienced a higher occurrence (48%) of CHD compared to the other race groups (31%) (Fig. 1b). Rates of CHD were lower among women with an ideal CVH score (24%) as compared to those with CVH at non-ideal levels (61.9%) (Fig. 1c). Receipt of potentially cardiotoxic breast cancer treatments was associated with an increased occurrence of post-treatment CHD. Rates of incident CHD were higher among women who received any cancer treatment (58.9%) compared to the women who did not receive any cancer treatments (29.1%) (Fig. 1d). We observed similar trends for the outcome of mortality (Fig. 1e-h). Particularly, Fig. 1f shows 29% of the black women died compared to 15.9% for the non-black race; Fig. 1h shows that higher percentage of patients died in the recipient of treatment group. Women who died during the 10-year follow-up tended to be older, of black race, who received cancer treatments, and who had non-ideal CVH.

**Fig. 2:**
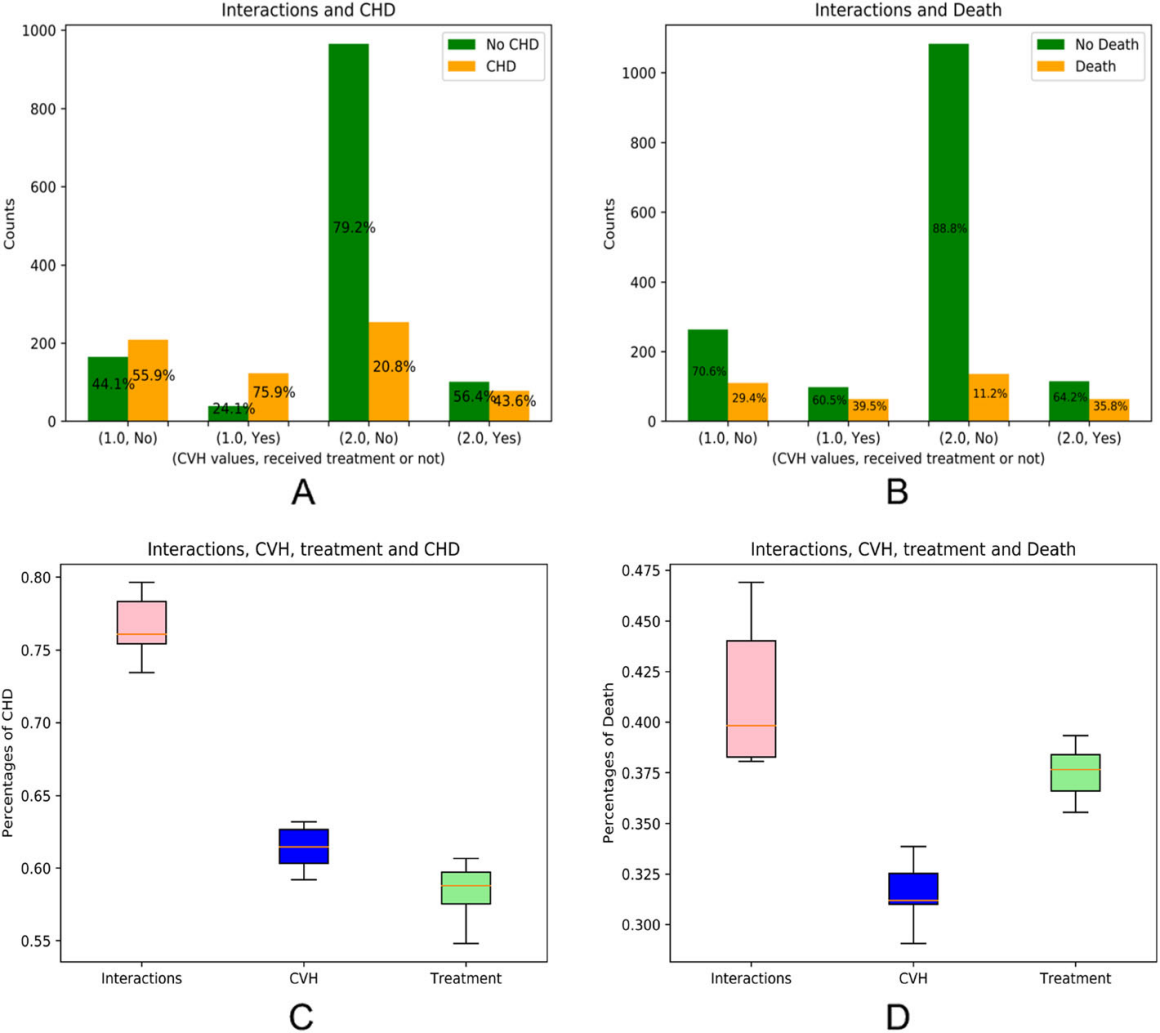
Associations between joint effects/interactions and CHD or death. **a** and **b** show the contribution of CVH and treatments on CHD (**a**) and death (**b**). **c** and **d** are box plots of the individual and joint (interaction) effects of CVH and treatments on CHD (**c**) and death (**d**). In **a** and **b**, CVH is ideal if CVH = 2.0, and intermediate if CVH = 1.0

In Fig. 2, we show the independent and joint effects of receipt of cardiotoxic breast cancer treatments and poorer CVH. Women in poor (non-ideal) CVH who were also exposed to cardiotoxic treatments had a synergistically higher occurrence of post-treatment CHD (75.9%) compared to women not exposed to cardiotoxic treatments who were in good CVH (20.8%) (Fig. 2a). Women in poor CVH who were not exposed to cardiotoxic treatments, as well as women in good CVH who were exposed to cardiotoxic treatments, had an elevated occurrence of post-treatment CHD (55.9 and 43.6% respectively), but did not experience a rate as high as those who were doubly-exposed. Similar trends were observed for the outcome of mortality (Fig. 2b). In addition, the independent effect of treatment is bigger on CHD (43.6%) than on death (35.8%).

The boxplots in Fig. 2c also indicate the significant difference between CHD rates among women who were doubly-exposed and the women who were independently exposed to poor CVH and cancer treatments (*p* < 0.0001 for poor CVH vs. joint exposure, *p* < 0.0001 for cancer treatments vs. joint exposure). We observed similar results for the outcome of mortality shown in Fig. 2d (*p* < 0.0001 for poor CVH vs. joint exposure, *p* < 0.007 for cancer treatments vs. joint exposure).

We obtained similar results using machine learning models. Table 3/4 (in the supplementary material) lists the performance results for death/CHD prediction by the three models. The accuracy for predicting death by SVM was 69% for models containing CVH (68% by decision tree, 69% by logistic regression), 63% for models containing cancer treatment (69% by decision tree, 66% by logistic regression) and 70% for models containing both CVH and treatment (72% by decision tree, 72% by logistic regression). The metrics of precision, recall and f1-score had a similar trend for the prediction. The prediction performance results held the same trends for the CHD prediction (Table 4).

**Fig. 3:**
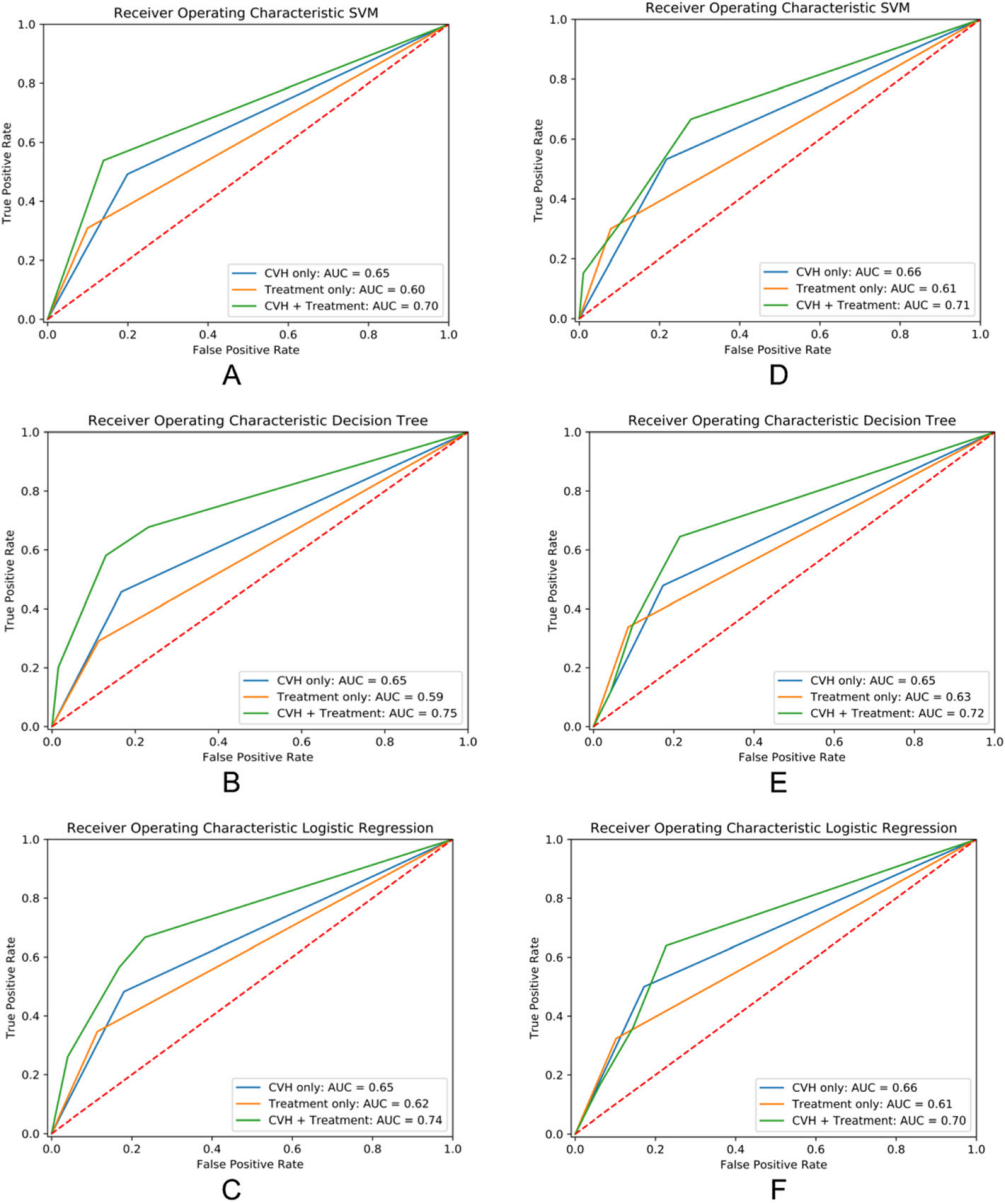
The first column (**a-c**) represent CHD prediction, and the second column (**d-f**) show results of mortality prediction. **a** and **d** show the AUC in ROC by SVM models, **b** and **e** show the AUC in ROC by decision tree models, and **c** and **f** show the AUC in ROC by logistic regression models. The three curves in each plot represent the individual and joint effects of CVH and potentially-cardiotoxic treatments

The first column in Fig. 3 shows the AUC plots for CHD prediction while the second column shows the prediction characteristics for the outcome of death by SVM, decision tree, and logistic regression classifiers under three different conditions of features: CVH, cancer treatments, and combined CVH and cancer treatments. The average AUC of the three machine learning models was 0.65 for CVH, 0.60 for cancer treatment, and 0.73 for both CVH and cancer treatments. We obtained similar results for the mortality analyses. We also performed 10-fold cross validation for each model and the results were similar (data not shown).

The results from Table 3 and Fig. 3 indicate that all three models achieved higher accuracy with the inclusion of joint effects as compared to only individual effects. Specifically, models which include both CVH and receipt of treatment data provide additional information and improve the prediction of CHD and death. Patients with poor overall CVH who received cancer treatments had the highest risk of CHD and death.

## Discussion

In this study, we utilized data from the EHR to identify women who were diagnosed with breast cancer in order to examine the independent and joint effects of CVH and cancer treatments on 10-year risk of post-treatment CHD or death. Our results indicated women with ideal CVH scores, and those who did not receive potentially cardiotoxic cancer treatments had the lowest risk of post-treatment CHD or death, while the joint effects of poor CVH and exposure to cancer treatments significantly increased the risk of post-treatment CHD or death. Additional factors that were associated with a higher prevalence of CHD and death included older age and black race.

Our results were consistent with previous conclusions that minority and older adults were more likely to have poorer CVH and ideal CVH was inversely associated with cancers and cardiovascular disease [20]. Consistent with biologic plausibility, our results indicated a higher risk of post-treatment CHD among those who received breast cancer treatments such as ionizing radiation to the heart [27].

The innovation in this study was to investigate the joint effects of CVH and potentially cardiotoxic breast cancer treatments by both statistical methods and multiple machine learning approaches. The results from all these methods were consistent, indicating the robustness of our methods and results.

Our next step is to investigate some questions such as which individual treatments (e.g. anthracyclines, hormone therapy) and individual CVH submetrics (e.g. BMI, blood pressure) are the most important variables for predicting CHD and death, but these questions are beyond the scope of this paper. We also plan to replicate these analyses in distinct cancer types.

Another next step is to involve deploying and evaluating clinical decision support in the cancer survivorship setting for managing cardiovascular late effects among cancer survivors. Our clinical decision support system (CDSS) presents CVH and cancer treatment data separately in the EHR-embedded data visualization. Our goal is to 1 day integrate a validated cardiovascular risk algorithm into our existing CDSS to better target cardiovascular disease prevention and management efforts in cancer survivorship.

## Limitations

We encountered many limitations in using EHR data for these analyses. First, there were many missing data for CVH, likely because these women were not being seen for preventive care but rather for cancer care and treatment. Second, we acknowledge that we may be missing CHD and mortality outcome data for women who obtained cancer care and treatment at our medical center but after which returned home and sought care outside of our medical center. Third, we used CVH measurements up to 5 years prior to the cancer diagnosis, of which the time frame varied for each woman. Fourth, physical activity and diet data are not commonly recorded in the EHR as structured, actionable data elements. If physical activity and diet data do exist in the EHR, they are usually recorded as clinical notes using free text. Importantly, these data are not easily translated into the American Heart Association’s metric definitions and thus are not actionable at the point-of-care or easily incorporated into risk scoring algorithms. Similarly, data on diagnosis and treatment, have not always been stored as structured data elements in the EHR. Conducting this analysis required mining data from legacy EHR systems and for pragmatic reasons we accessed only structured data elements for this analysis, which resulted in incomplete data ascertainment. Finally, we are missing data on radiotherapy. We will explore the missing radiotherapy data from multiple data sources as our future work.

## Conclusions

An ideal CVH score predicted a lower risk of post-treatment CHD or death. Receipt of cardiotoxic breast cancer treatments was associated with increased post-treatment CHD or death, and there was a synergistic effect of CVH such that better CVH seemed to be protective against the development of CHD even among women who had received potentially cardiotoxic treatments. This study determined the extent to which ideal CVH is important to attain and maintain for more favorable outcomes following a breast cancer diagnosis.

## Supplementary information

**Additional file 1 Table S1** Performance results of the three models for prediction of the outcome of mortality. **Table S2** Performance results of the three models for prediction of the outcome of CHD.

## Data Availability

The datasets used during the current study are available from the corresponding author on reasonable. request.

## Abbreviations

CHD: Coronary heart disease
CVH: Cardiovascular health
AHA: American Heart Association
EHR: Electronic health record
BMI: Body mass index
AUC: Area under the receiver operator curve
SVM: Support vector machines
