# Supplementary material for: Coronary heart disease and mortality following a breast cancer diagnosis
Source: BMC Med Inform Decis Mak. 2020 May 13;20:88. doi: 10.1186/s12911-020-1127-y (PMC7218836; doi:10.1186/s12911-020-1127-y)
Supplement: Supplementary file 1 — Additional file 1 Table S1 Performance results of the three models for prediction of the outcome of mortality. Table S2 Performance results of the three models for prediction of the outcome of CHD. [file 12911_2020_1127_MOESM1_ESM.docx]

**Table S1. Performance results of the three models for prediction of the outcome of mortality.**

| Models | SVM | | | Decision Tree | | | Logistic Regression | | |
| --- | --- | --- | --- | --- | --- | --- | --- | --- | --- |
| Metrics | CVH | treatment | interaction | CVH | treatment | interaction | CVH | treatment | interaction |
| Accuracy | 0.69 | 0.63 | **0.70** | 0.68 | 0.69 | **0.72** | 0.69 | 0.66 | **0.72** |
| Precision | 0.67 | 0.68 | **0.70** | 0.68 | 0.70 | **0.72** | 0.69 | 0.67 | **0.71** |
| Recall | 0.67 | 0.63 | **0.70** | 0.68 | 0.69 | **0.72** | 0.69 | 0.66 | **0.72** |
| F1-score | 0.66 | 0.59 | **0.70** | 0.67 | 0.66 | **0.72** | 0.68 | 0.62 | **0.71** |

**Table S2. Performance results of the three models for prediction of the outcome of CHD.**

| Models | SVM | | | Decision Tree | | | Logistic Regression | | |
| --- | --- | --- | --- | --- | --- | --- | --- | --- | --- |
| Metrics | CVH | treatment | interaction | CVH | treatment | interaction | CVH | treatment | interaction |
| Accuracy | 0.69 | 0.68 | **0.74** | 0.70 | 0.69 | **0.77** | 0.69 | 0.69 | **0.73** |
| Precision | 0.58 | 0.56 | **0.70** | 0.70 | 0.69 | **0.77** | 0.69 | 0.69 | **0.73** |
| Recall | 0.58 | 0.57 | **0.71** | 0.72 | 0.72 | **0.78** | 0.70 | 0.71 | **0.74** |
| F1-score | 0.58 | 0.56 | **0.71** | 0.70 | 0.69 | **0.77** | 0.69 | 0.69 | **0.73** |

The metrics of accuracy, precision, recall and f1-score were calculated by the following equations:

$$Accuracy=(TP+TN)/(TP+TN+FP+FN)$$

$$Precision=TP/(TP+FP)$$

$$Recall=TP/(TP+FN)$$

$$F1 score=2/((1/Precision)+(1/Recall))$$
